# A rare case of retained sabot after close-range shotgun injury

**DOI:** 10.1186/s40792-021-01238-z

**Published:** 2021-06-24

**Authors:** J. Alford Flippin, Sami Kishawi, Hannah Braunstein, Alaina M. Lasinski

**Affiliations:** 1 Department of Surgery, Division of Trauma, Critical Care, Burns, and Acute Care Surgery, MetroHealth Medical Center, 2500 MetroHealth Drive, Cleveland, OH 44109-1998 USA; 2 Case Western Reserve University School of Medicine, Cleveland, OH USA

**Keywords:** Shotgun, Sabot, Wadding, Retained foreign object, Case report

## Abstract

**Background:**

Shotgun injuries are a relatively uncommon type of trauma, and therefore may present a challenge in management for trauma surgeons. This is particularly true in the case of surgeons unfamiliar with the unique characteristics of shotgun wounds and the mechanics of shotguns. In many cases, the shot pellets are the primary source of injury. However, a broad understanding of shotgun mechanics is important in recognizing alternative presentations. This article details a case of sabot (a stabilization device used with certain projectiles) retention after a close-range shotgun injury, reviews underlying shotgun mechanics, and discusses strategies for the detection and mitigation of these injuries. The aim of this case report is to increase awareness of and reduce the potential morbidity of close-range shotgun injuries.

**Case presentation:**

A middle-aged female presented to the Emergency Department with wounds to her right hip and flank after suffering a shotgun injury. A contrast computed tomography scan demonstrated no evidence of hollow viscous or vascular injury, but was otherwise severely limited by scatter artifact from the numerous embedded pellets. The patient was admitted for wound care and discharged 2 days later with a clean wound bed and no evidence of tissue necrosis. Six days after injury, she reported an “unusual” smell associated with severe pain in her right hip wound. She was evaluated in clinic where examination revealed a retained foreign body, identified to be a shotgun shell sabot, which was removed in clinic. She presented again several days before scheduled follow-up with a persistent foul smell from her wound and was noted to have necrotic tissue at the base and margins of the wound that required hospital readmission for operative debridement and closure with negative pressure wound therapy. The patient had an uncomplicated recovery after surgical debridement.

**Conclusions:**

Although shotgun sabot penetration and retention are rare, they are associated with significant morbidity. Sabot penetration should be considered if injury narrative, physical examination, or radiographic characteristics indicate a distance from shotgun to patient of less than 2 m. A high degree of suspicion is indicated at less than 1 m.

## Background

Injury by shotgun is a relatively uncommon form of trauma despite the large number of these weapons worldwide and the less restrictive environment surrounding their ownership [1]. For this reason, trauma surgeons may be unfamiliar with shotgun mechanics and the unique characteristics of shotgun-induced wounds, especially those sustained at close range, and the potential pitfalls of their management.

**Fig. 1:**
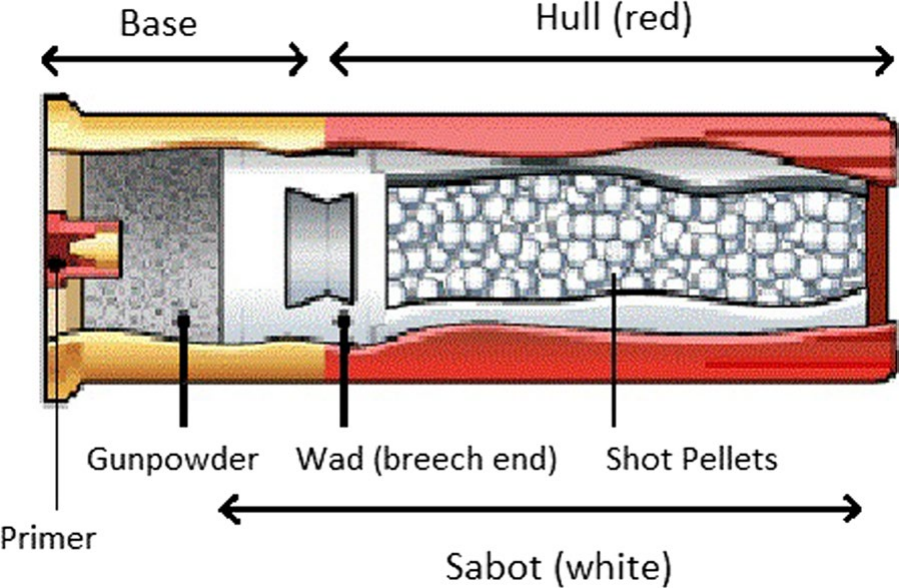
A cut-away diagram demonstrating the components of a shotgun shell. A brass base (yellow) and hard plastic hull (red) compose the exterior. Inside the base are the primer and gunpowder. Inside the hull are the shot pellets, themselves contained in the sabot (white), the breech end of which is called a wad

Shotgun shells are composed of a brass base which holds propellant gunpowder bonded to a hard-plastic shell case called a hull. This hull contains a softer plastic “wad” which is solid at its breech end and hollow at its barrel end to contain the pellets (collectively, “shot”) characteristic of shotguns (Fig. 1) [2]. This wadding is a form of ballistic sabot, a structural device which keeps a projectile (or projectiles, in the case of shotgun pellets) with a significantly smaller diameter than the barrel centered therein. Upon exiting the barrel, the sabot fractures under the pressure differential and releases the shot contained within it into a characteristic cone. The effective range is rarely more than 35 to 45 m unless loaded with non-pellet ammunition. Once the sabot exits the barrel and no longer contains shot, it has a large surface area-to-mass ratio and rarely travels more than 2 m beyond the end of the barrel [3].

In this article, we will discuss the unique characteristics of shotgun wounds; present an interesting case of a close-range shotgun injury; and, novel to the literature, discuss methods to detect and mitigate complications related to these injuries. The aim of this case report is to increase awareness of and reduce the potential morbidity of close-range shotgun injuries.

## Case presentation

**Fig. 2:**
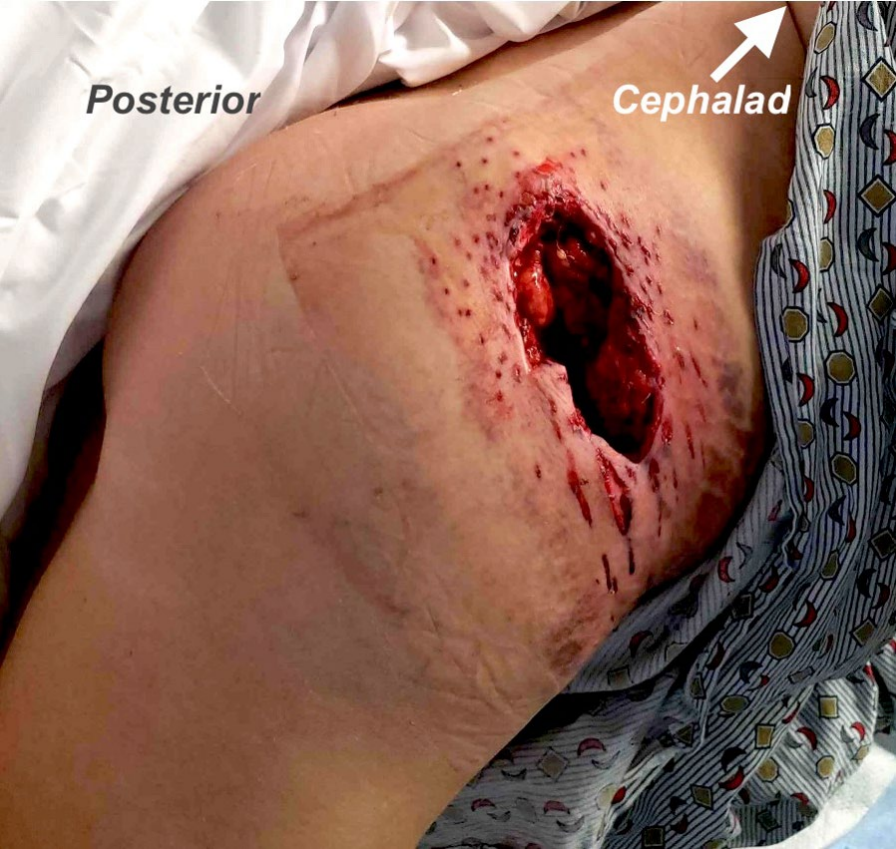
Image of the patient’s wound on the day of injury

A middle-aged female patient was brought to our hospital as a category 1 (the most critical level) trauma activation after being struck in the right hip and flank by a shotgun discharge during an episode of interpersonal violence. Upon arrival, she was found to have a hemostatic 8-cm × 5-cm irregular wound to the right flank and hip tracking medially and deep, with exposure of muscle (Fig. 2). Primary and secondary surveys revealed no other traumatic injuries and she was hemodynamically normal.

Her workup included a computed tomography (CT) scan of her abdomen and pelvis with intravenous (IV) and rectal contrast to evaluate for peritoneal violation and hollow viscus injury. While the imaging was seriously limited by the scatter artifact of several hundred shotgun pellets tightly grouped into a small area, it was observed that none had entered the peritoneal cavity and there was no contrast extravasation from the rectum or other evidence of hollow viscus or vascular injury. Due to the large area of tissue destruction from the shotgun injury and the radiolucent nature of the plastic shotgun sabot, it was not apparent on CT that a foreign body was present other than the shotgun pellets. As discussed further in the next section, this large pocket of destroyed tissue, now filled with air, serves as both a clue that a sabot may be present and a confounder because that sabot is radiolucent.

The patient was admitted for wound care and physical therapy given the severely limited range of motion in her right lower extremity. The patient’s wound care regimen included an initial washout with betadine and saline following which it was packed with a gauze roll and covered with gauze. This dressing was to be changed daily. Given the number of pellets and their dispersal in the soft tissues, it would not have been practical to remove them without significant tissue disruption. Over the next 2 days, her wound was packed with saline wet-to-dry dressings daily and appeared healthy. She showed progress with mobility such that she was cleared for discharge 2 days after her injury.

**Fig. 3:**
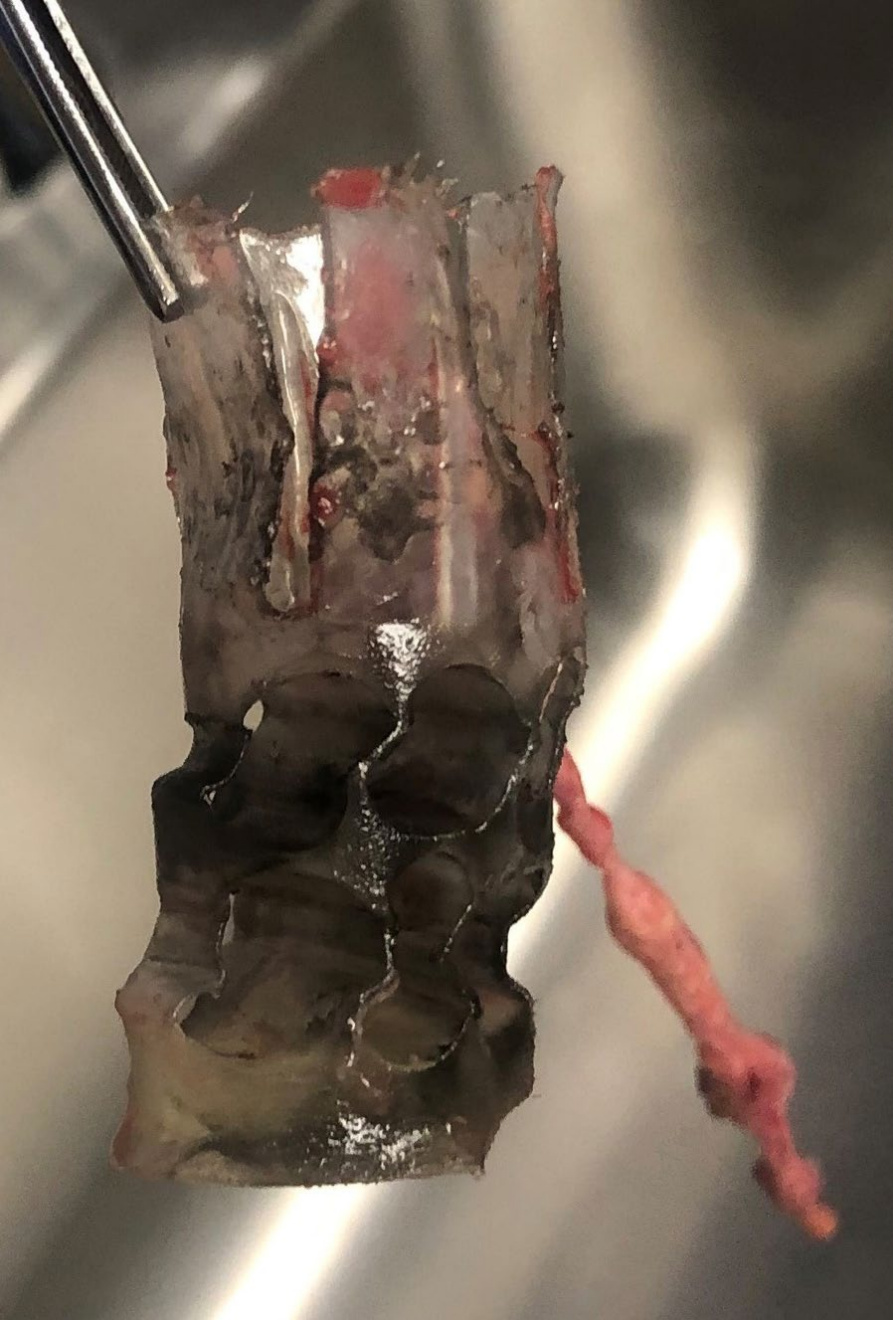
The sabot removed from the patient

Six days post-injury, she requested to be seen in clinic after noting an “unusual” smell and developing severe pain at the site of the injury. Upon examination, a foreign body was observed embedded deep in the medial margin of the wound, with the shallowest edge 2–3 cm below the surface of the wound and the odor of gunpowder was recognized. It was only at this time that the distance to the weapon upon discharge, less than one meter, was elicited specifically from the patient. With minor difficulty, the object was mobilized and removed in the clinic. It was immediately recognized as a shotgun shell sabot (Fig. 3) and turned over to Pathology for forwarding to law enforcement per our foreign body protocol. At this time, she was noted to have some areas of threatened skin and subcutaneous tissue, but nothing requiring immediate debridement. She was instructed to continue daily wet-to-dry dressing changes and was scheduled for close follow-up.

She returned several days later, prior to scheduled follow-up, with renewed concerns about her wound. Upon evaluation, the wound was noted to have necrotic tissue at its base and skin margins. She was admitted to the hospital and underwent surgical debridement with eventual closure with negative pressure wound therapy. She had an uncomplicated course following this operation.

## Discussion and conclusions

As evidenced by our case, as well as others cited, shotgun sabot penetration and retention are rare occurrences, but carry risk for significant morbidity. In general, the shot pellets are the main cause of injury. However, several cases have been reported in which the injury happened at close range and the sabot was also ejected into the patient [4]. In one case, the sabot was ejected into the chest cavity causing delayed pulmonary cavitation and massive hemoptysis [5]. In another, the sabot penetrated the gastric lumen causing delayed reflux of the sabot into the esophagus and subsequent esophageal perforation [6].

Similar to other projectiles, the pellets themselves are not a significant risk factor for wound infection, so it is our practice not to routinely remove them unless there is another specific indication for removal such as a foreign body in a joint space, causing nerve impingement, or in the lumen of a vessel or spinal canal [7, 8]. We do not routinely give more than a single dose of antibiotics, usually a first-generation cephalosporin, except in the case of a fracture [9].

Shotgun injuries and their associated sequelae often require more operations and resource utilization compared to other firearm injuries [10]. Detection requires knowledge of shotgun mechanics as well as a high degree of vigilance in the examination and management of the patient.

Fortunately, information can be drawn from the injury narrative, examination of the wound, and radiographic findings which serve to raise suspicion for sabot ejection into a wound. If the patient or a bystander can provide an injury narrative, the distance from the shotgun to the victim is key information. Any distance less than 2 m should raise suspicion, and a distance less than 1 m should be considered high risk for retained sabot. In our patient, the exact distance was not specifically obtained until the first clinic visit. It is possible that obtaining this element of the history with greater specificity earlier in the clinical course may have allowed earlier identification of the sabot in the wound.

Physical examination can also give clues that a shotgun injury was sustained at a close enough range that sabot ejection into the wound is possible or even likely. Our patient’s wound contained a large contiguous area of tissue loss, as opposed to more widely spaced puncture wounds from individual pellets, suggesting that insufficient distance existed between the shotgun and the victim to allow for shot to spread. This feature should trigger the trauma provider to suspect a heightened chance that the sabot followed the shot into the wound.

**Fig. 4:**
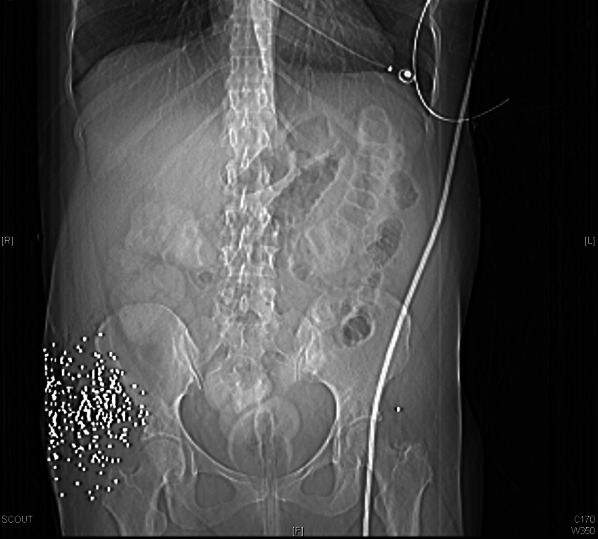
Abdominal X-ray showing numerous shot pellets contained in a relatively small area near the patient’s right hip

Finally, radiographic characteristics can help stratify patients for clinical concern. Indeed, formulae exist to approximate the distance from shotgun to victim, but in the acute setting, these are prohibitively time consuming to be useful to the traumatologist [3]. More usefully, rapid generalizations can be made based on plain X-ray or CT scan of the affected area. If large numbers of shot pellets are contained in a small area, as in our patient (Fig. 4), this indicates that the shot had insufficient range to spread into a wider cone, suggesting that higher suspicion for sabot penetration is warranted.

In conclusion, when evaluating a victim of a shotgun injury, there are key elements in a patient’s history, physical examination, and radiography that should raise clinical suspicion for a retained shotgun sabot. In our case, elements of the history, physical exam, and radiographic findings all pointed to a high risk for retained shotgun sabot. It is possible that had these factors been considered and the index of suspicion higher, the sabot may have been located at the initial wound exploration or during subsequent dressing changes. In all cases, this increased suspicion may lead to decreased morbidity and mortality for the patient and cost for the healthcare system.

While the patient described in this case report provided consent for us to publish this report, she understandably chose not to provide her perspective regarding her injury and clinical course, as it was sustained during an episode of interpersonal violence.

## Data Availability

The relevant data and images related to the patient’s course and care are included in the article.
